# Peroral Cholangioscopy-Guided Forceps Mapping Biopsy for Evaluation of the Lateral Extension of Biliary Tract Cancer

**DOI:** 10.3390/jcm10040597

**Published:** 2021-02-05

**Authors:** Takumi Onoyama, Wataru Hamamoto, Yuri Sakamoto, Shiho Kawahara, Taro Yamashita, Hiroki Koda, Soichiro Kawata, Yohei Takeda, Kazuya Matsumoto, Hajime Isomoto

**Affiliations:** 1 Division of Gastroenterology and Nephrology, Department of Multidisciplinary Internal Medicine, Faculty of Medicine, Tottori University, Nishi-cho 36-1, Yonago 683-8504, Japan; hamamoto_trr@yahoo.co.jp (W.H.); yuri.sakamoto@mac.com (Y.S.); kawahara.hp1@gmail.com (S.K.); yamat11@gmail.com (T.Y.); po.polnga.3.negaiwo.xxx@gmail.com (H.K.); kawataso0527@yahoo.co.jp (S.K.); yhytkd7@outlook.jp (Y.T.); isomoto@tottori-u.ac.jp (H.I.); 2 Internal Medicine, Irisawa Medical Clinic, Yawata-cho 285-6, Matsue 690-0025, Japan; matsumotokazuya@tottori-u.ac.jp

**Keywords:** peroral cholangioscopy, mapping biopsy, biliary tract cancer, lateral extension, superficial intraductal spread

## Abstract

Background: Peroral cholangioscopy (POCS)-guided forceps mapping biopsy (FMB) is a method for the accurate preoperative identification of the extent of the disease of biliary tract cancer (BTC). However, the diagnostic value of POCS-FMB is still uncertain. Objectives: We evaluated the diagnostic utility of POCS-FMB for the identification of lateral extension—superficial intraductal spread longitudinally and continuously from the main lesion—of BTC. Methods: In the retrospective study, patients who received POCS-FMB and surgery for curative resection of BTC between September 2016 and August 2019 at our medical institution were enrolled. The diagnostic accuracy of POCS-FMB for the identification of lateral extension of BTC was evaluated. Furthermore, we also evaluated the factors affecting the diagnostic accuracy of POCS-FMB. Results: A total of 23 patients with BTC were enrolled, and 24 procedures of POCS-FMB from 96 sites of biliary tracts were performed. The sensitivity, specificity, and accuracy of POCS-FMB were 53.8%, 63.9%, and 63.1%, respectively. In the multivariate logistic regression analyses, the biopsy from the bifurcation of biliary tracts was a significant factor affecting the diagnostic accuracy of POCS-FMB (odds ratio 3.538, 95%; confidence interval 1.151–10.875, *p* = 0.027). Conclusions: The diagnostic accuracy of POCS-FMB for the identification of lateral extension of BTC was insufficient. The biopsy from the bifurcation of biliary tracts was a positive factor affecting the diagnostic accuracy of POCS-FMB.

## 1. Introduction

Biliary tract cancer (BTC) is among the poor prognostic malignant diseases, with a low 5-year survival rate (18.7%) and short survival time (median, 8.5 months) [1,2,3]. To improve the prognosis of BTC, early diagnosis of BTC is most important. However, it is difficult to distinguish BTC from benign biliary strictures, such as immunoglobulin G subclass 4 (IgG4)-associated sclerosing cholangitis, and primary sclerosing cholangitis [4]. Therefore, a pathological diagnosis sampled by endoscopic techniques is desirable before any invasive treatment. Although the specificity of endoscopic retrograde cholangiopancreatography (ERCP)-related tissue sampling for indeterminate biliary lesions is high, the sensitivity is insufficient [5,6]. The highly desmoplastic nature of BTC may limit the accuracy of cytological and pathological diagnosis. Meanwhile, genomic analyses, such as liquid biopsy, have been applied for the diagnosis of BTC [7,8]. Therefore, it is controversial whether the pathological diagnosis is or is not necessary, especially for patients with potentially resectable lesions. On the other hand, curative surgical resection is also important to improve the prognosis of BTC [3,9,10]. However, it is sometimes difficult to perform curative resection for BTC. Superficial intraductal spread is a clinical feature of BTC in which the atypical epitheliums extend longitudinally and continuously from the main lesion. This feature is present in 14.6% of patients with cholangiocarcinoma [9,11]. The presence of lateral extension results in non-curative surgical resection for BTC. Therefore, an exact evaluation of perihilar and distal margins of preoperative BTC is not only essential for assessing resectability but also for determining the appropriate resection line. Furthermore, surficial intraductal spread was shown in both patients with perihilar cholangiocarcinoma (11.4%) and patients with distal cholangiocarcinoma (25.0%) [11]. Therefore, to perform curative resection for BTC, an exact evaluation of lateral extension is not only required for perihilar cholangiocarcinoma but also distal cholangiocarcinoma. Although imaging findings including computed tomography (CT) and magnetic resonance imaging (MRI) are useful modalities for the assessment of intraductal spread of BTC [12], their diagnostic performances, especially specificities, were insufficient because it is difficult to distinguish BTC from inflammatory changes [13]. Needless to say, a liquid biopsy is not useful for the evaluation of the lateral extent of BTC. Transpapillary forceps mapping biopsy (FMB) in fluoroscopy, one of the ERCP techniques, has been used for the identification of lateral extension of BTC [14]. Recently, some studies have reported the usefulness of peroral cholangioscopy (POCS)-guided forceps biopsy for malignant biliary diseases [15,16]. Moreover, previous reports have also revealed the usefulness of POCS-FMB for the preoperative identification of the lateral extension of BTC [17,18]. Meanwhile, Nishikawa et al. reported that the diagnostic accuracy of the POCS visual findings for lateral extension of BTC was higher than that of the POCS-FMB [19]. Although POCS plays an important role in the preoperative evaluation of BTC, the diagnostic utility of POCS-FMB is still uncertain. There is no study showing the diagnostic performance of POCS-FMB for the identification of lateral extension of BTC by the biopsy site, especially.

The objective of the study was to evaluate the diagnostic ability of POCS-FMB for the identification of lateral extension of BTC. Furthermore, the factors affecting the diagnostic accuracy of POCS-FMB were also evaluated.

## 2. Materials and Methods

### 2.1. Study Population

Patients who received ERCP-related tissue sampling with POCS for biliary disease between September 2016 and August 2019 at our medical institution were retrospectively enrolled. Inclusion criteria were as follows: (1) Patients who received POCS-FMB to identify lateral extension of BTC; (2) Patients who underwent surgical resection for BTC; (3) Patients aged 20 years or older when the POCS-FMB were performed. Exclusion criteria were as follows: (1) Patients who did not provide consent; (2) Patients who did not receive surgical resection for BTC; (3) Patients who received neoadjuvant chemotherapy for preoperative BTC.

**Figure 1:**
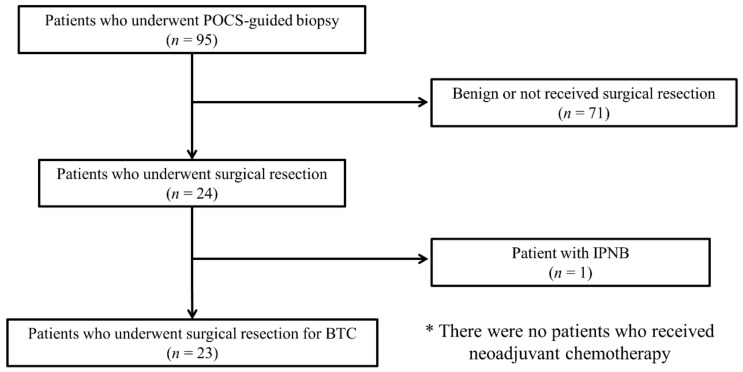
Flowchart of selected patients in the study. Abbreviations: POCS, peroral cholangioscopy; BTC, biliary tract cancer; IPNB, intraductal papillary neoplasm of the bile duct.

In this study, 23 patients with BTC were enrolled (Figure 1). Participants included 16 men and 7 women aged 56–84 years (median age, 72 years) (Table 1).

### 2.2. Endoscopic Procedure

In the study, we performed ERCP with a side-viewing duodenoscope (JF260V/TJF240V/TJF290V, Olympus Corp., Tokyo, Japan). POCS was performed by using a mini endoscope (M00546600 SpyGlass DS Access; Boston Scientific, Marlborough, MA, USA). A cholangioscope was inserted into the bile duct over a 0.025-inch hydrophilic guidewire (G-260-2545A; Olympus Corp., Tokyo, Japan; MTA0025N48S; Medico’s Hirata Inc, Osaka, Japan; M00556700; Boston Scientific Corporation, Marlborough, MA, USA), and M00546270 (SpyBite Biopsy Forceps; Boston Scientific, Marlborough, MA, USA) was used for POCS-FMB under direct vision.

Endoscopic sphincterotomy (EST) by using a sphincterotome (Olympus CleverCut KD-V411M-0725; Olympus Corp., Tokyo, Japan) was performed for almost all patients who had no history of EST. If patients had received antithrombotic therapy, endoscopic papillary balloon dilation (EPBD) using a balloon dilatation catheter (ZR25-08-23, RN25-0630-18; KANEKA Medix Corporation, Osaka, Japan) was performed to insert a cholangioscope into the biliary system.

The FMB was defined as the tissue acquisition of the non-stenotic bile duct without caliber changes in the biliary tract diameter in fluoroscopy images of ERCP. The location of POCS-FMB included distal bile ducts, a confluence of cystic ducts, perihilar bile ducts, a confluence of the right and left hepatic ducts, right hepatic ducts, left hepatic ducts, a confluence of the anterior and posterior segmental ducts, B4 confluence, a confluence of B5 and B8 segmental ducts, a confluence of B6 and B7 segmental ducts, and a confluence of B2 and B3 segmental ducts.

### 2.3. Diagnostic Criteria of POCS-Guided Forceps Mapping Biopsy

**Figure 2:**
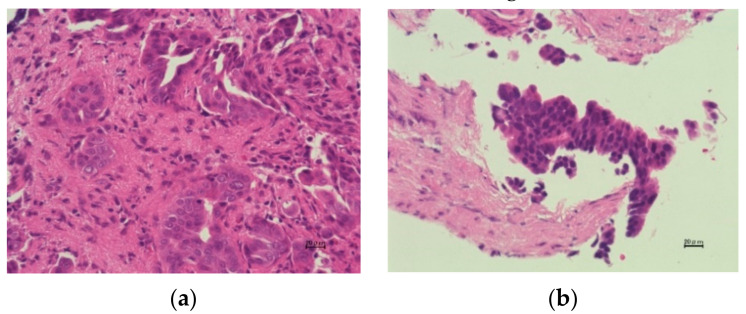

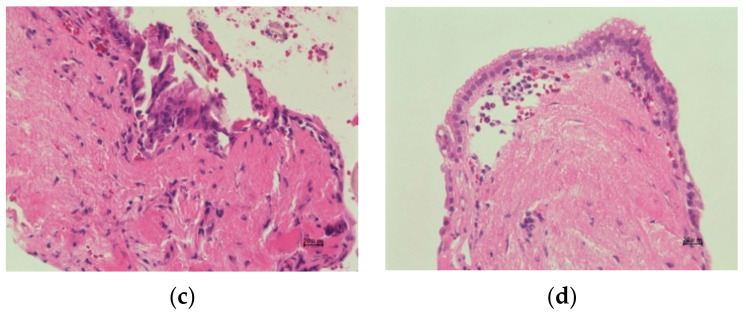
Histopathological findings of specimens obtained via peroral cholangioscopy-guided forceps mapping biopsy. (**a**) Biliary epithelial cells with severe cellular and structural atypia are revealed (adenocarcinoma) (**b**) Biliary epithelial cells with severe atypia are revealed (biliary intraepithelial neoplasia-3). (**c**) Biliary epithelial cells with mild atypia are shown (biliary intraepithelial neoplasia-1). (**d**) Normal biliary epithelial cells are revealed. Scale bar = 20 μm

Biopsy specimens were stained with hematoxylin and eosin. Immunohistochemistry, including p53 and Ki-67, was also performed as needed. Malignancy or suspected malignancy including biliary intraepithelial neoplasia-3 was considered positive in histopathological findings. Benign and mild atypia were considered negative (Figure 2). We defined indeterminate in histopathological findings as misdiagnosis. Specimens that did not include epithelium were also considered as misdiagnosis.

Diagnostic accuracy of the lateral extension of BTC was defined as corresponding with the tumor extent confirmed by the histopathological examination of resected specimens. The biopsy sites that were not included in the excision range were evaluated by surgical margin as positive or not. If the surgical margin was negative, the biopsy sites outside the surgically resected specimen were considered benign. Meanwhile, the biopsy sites outside the resected specimen could not be evaluated in the cases with positive surgical margin. In sum, the final diagnosis was defined as malignancy if the local recurrence appeared in the biopsy site within 6 months after surgery.

### 2.4. Statistical Analysis

The factors affecting the diagnostic accuracy of POCS-FMB were also evaluated. By using StatFlex ver. 7.0 for Windows (Artec Corp., Osaka, Japan), statistical analysis was performed. All values are expressed as median with interquartile range. Subgroup analyses of age (<75 or ≥75 years), sex, the location of the biliary lesion or stricture (distal or non-distal), length of biliary stricture (<15 or ≥15 mm), macroscopic types of BTC (flat type or non-flat type composed of nodular and papillary type), histological differentiation of BTC (tubular adenocarcinoma or not differentiation composed of the papillary and poor differentiation), T category (<T3 or ≥T3), biopsy site (bifurcation of the bile duct or not, intrahepatic bile duct or extrahepatic bile duct, right side or left side), the presence of acute cholangitis, level of serum T-Bil (<1.5 or ≥1.5 mg/dL), level of CEA (<5.0 or ≥5.0 ng/mL), level of CA19-9 (<35 or ≥35 U/mL), procedure time (≤75 or >75 min), EST, and previous biliary stenting before POCS-FMB were assessed to determine the diagnostic accuracy of POCS-FMB for lateral extension of BTC. Univariate analyses were carried out to assess the diagnostic accuracy of the disease range of BTC, and factors with *p* < 0.1 were included in the multivariate logistic regression analyses. *p* <0.05 was considered to be a significant difference.

## 3. Results

### 3.1. Patients’ Characteristics and Baseline Evaluation

Table 1 showed the characteristics of patients with BTC. One patient had two lesions of BTC in his perihilar and distal bile duct. Therefore, the study group included 16 lesions in the distal bile duct, 4 lesions in the perihilar bile duct, 3 lesions in the cystic duct, and 1 lesion in the gallbladder, respectively. Macroscopic types of BTC included 5 papillary-type, 13 nodular-type, and 6 flat-type. Histological differentiation of BTC included 12 lesions with well-differentiated tubular adenocarcinoma, 7 with moderately differentiated tubular adenocarcinoma, 3 with poorly differentiated adenocarcinoma, and 2 with papillary adenocarcinoma. The median length of biliary stricture was 16.8 mm (range, 4.8–45.6 mm). The median levels of serum total bilirubin (T-Bil), carcinoembryonic antigen (CEA), and carbohydrate antigen 19-9 (CA19-9) were 1.6 mg/dl (range, 0.5–14.8 mg/dL), 2.6 ng/mL (range, 0.8–8.3 ng/mL), and 35.2 U/mL (range, 6.4–9052.0 U/mL), respectively. Table 1 also shows the TNM classification and pathological stage of BTC by lesion site. Five patients had lymph node metastasis. Of the 23 patients, 14 (60.9%) underwent pancreatoduodenectomy (PD), 4 (17.4%) underwent hepatectomy with extrahepatic bile duct resection, and 4 (17.4%) received extrahepatic bile duct resection with cholecystectomy. Hepatectomy and PD were performed for 1 patient (4.3%) with perihilar and distal cholangiocarcinoma.

Twenty-four procedures were performed since one patient received POCS-FMB 2 times (Table 2). The median procedure time of ERCP with POCS-FMB was 85 min (range, 57–124 min). EST was performed for 14 patients. Seven cases had previously undergone EST. Three times EPBD was performed for two patients who received antithrombotic therapy. POCS-guided forceps biopsy for biliary lesion was also performed for 19 patients in the same procedure, and 17 patients were diagnosed with adenocarcinoma (sensitivity, 89.5%).

The median follow-up period after surgical resection for BTC was 12 months (range, 2–35 months). During the follow-up period, 19 patients survived and 15 of them had no recurrence of BTC. Three patients died, two of whom were due to the recurrence of BTC. One patient who had a recurrence of BTC was transferred to the other hospital after 20 months of follow-up. In six patients with positive surgical margin, three of them received radiation therapy and one received adjuvant chemotherapy.

The total number of POCS-FMB sites was 96 in this study. The median number of biopsies was 2 (range, 1–4). POCS-FMB was performed at distal bile ducts in 8 patients, a confluence of cystic ducts in 8 patients, perihilar bile ducts in 3 patients, a confluence of the right and left hepatic ducts in 23 patients, right hepatic ducts in 5 patients, left hepatic ducts in 9 patients, a confluence of the anterior and posterior segmental ducts in 13 patients, B4 confluence in 9 patients, a confluence of B5 and B8 segmental ducts in 6 patients, a confluence of B6 and B7 segmental ducts in 2 patients, and a confluence of B2 and B3 segmental ducts in 10 patients. Biopsy specimens sampled from 21 biopsy sites could not be evaluated because they did not include epithelium; therefore, the adequate tissue acquisition rate was 78.1% (75/96).

**Figure 3:**
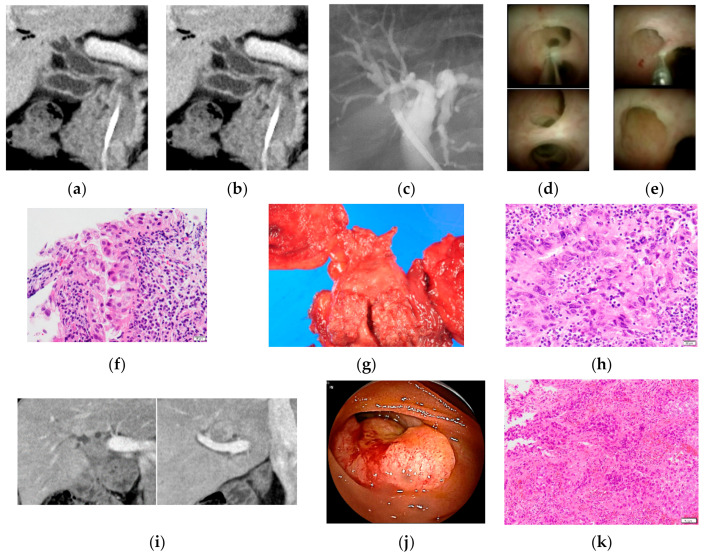
A case with local recurrences occurred 4 months after pancreatoduodenectomy for distal cholangiocarcinoma. (**a**,**b**) Computed tomography scan and magnetic resonance cholangiopancreatography revealed an irregular nodule and stenosis in the distal bile duct; (**c**) endoscopic retrograde cholangiography revealed no stenosis in the perihilar bile duct; POCS showed no irregular change in (**d**) confluence of B5 and B8 segmental ducts and (**e**) confluence of the anterior and posterior segmental ducts and POCS-guided forceps mapping biopsy was performed from them; (**f**) hematoxylin and eosin staining showed adenocarcinoma in specimens obtained from confluence of B5 and B8 segmental ducts and confluence of the anterior and posterior segmental ducts, Scale bar = 20 μm; (**g**) this patient underwent pancreatoduodenectomy; and (**h**) this patients was diagnosed with distal cholangiocarcinoma with positive surgical margin, Scale bar = 20 μm; (**i**) computed tomography scan revealed irregular nodules in confluence of B5 and B8 segmental ducts and confluence of the anterior and posterior segmental ducts 4 months after pancreatoduodenectomy; (**j**) balloon-assisted endoscopy revealed the local recurrence of the choledochojejunostomy; (**k**) hematoxylin and eosin staining revealed adenocarcinoma in specimens obtained from the choledochojejunostomy, Scale bar = 50 μm.

**Figure 4:**
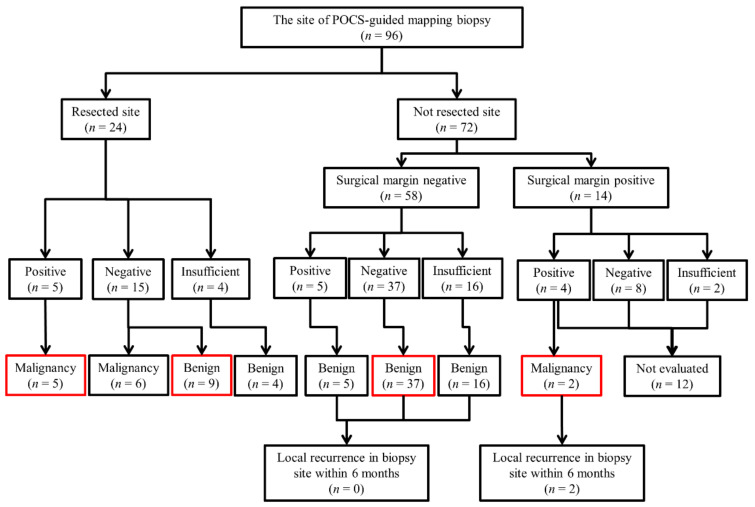
Diagnostic flowchart of POCS-guided mapping biopsy to identify lateral extension of BTC in the study. The final diagnosis in the red boxes was consistent with the POCS-guided forceps mapping biopsy results. Abbreviations: POCS, peroral cholangioscopy; BTC, biliary tract cancer.

Twenty-four biopsy sites were included in surgical resection. The number of biopsy sites outside the resected specimen was 72. The biopsy sites not included in the excision range with negative surgical margin were 58 and were defined as negative because there was no local recurrence in the biopsy site within 6 months. In patients with positive surgical margin, 14 of the biopsy sites were not included in the surgically resected specimens, and 2 of them were diagnosed as malignancy eventually, since local recurrences occurred 4 months after surgical resection (Figure 3). Therefore, 84 of the biopsy sites were evaluated for the diagnostic performance of POCS-FMB for the evaluation of lateral extension of BTC (Figure 4).

### 3.2. Diagnostic Performance of POCS-Guided Forceps Mapping Biopsy

Table 3 summarizes the diagnostic performance of POCS-FMB to identify the ranges of BTC by the biopsy sites. The values for sensitivity, specificity, and accuracy of POCS-FMB were 53.8%, 63.9%, and 63.1%, respectively (Table 3). In the site where the mapping biopsy resulted positive, seven of them were malignant, five were benign, and two could not be evaluated (positive predictive value; 58.3%, 7/12). Meanwhile, in the site where the biopsy resulted negative, 6 of them were malignant, 46 were benign, and 8 were difficult to evaluate (negative predictive value; 88.5%, 46/52) (Figure 4). The values for sensitivity, specificity, and accuracy of POCS-FMB for the extrahepatic bile duct were 45.5% (5/11), 62.9% (22/35), and 58.7% (27/46), respectively. Meanwhile, sensitivity, specificity, and accuracy of POCS-FMB for the assessment of lateral extension in the intrahepatic bile duct were 100% (2/2), 72.7% (24/33), and 74.3% (26/35), respectively. The values for sensitivity, specificity, and accuracy of POCS-FMB for bifurcation of the bile duct were 66.7% (6/9), 70.2% (40/57), and 69.7% (46/66), respectively.

### 3.3. Factors Affecting the Accuracy of POCS-Guided Forceps Mapping Biopsy

Table 4 reveals the result of analyses for the accuracy of POCS-FMB to identify the ranges of BTC. In the univariate analyses, the location of BTC, EST, and biopsy site (bifurcation of biliary tract) were candidate factors with *p* < 0.1, respectively, and were included in the multivariate logistic regression analyses. Biopsy site (bifurcation of biliary tract) was the significant factor affecting the diagnostic accuracy of POCS-FMB to identify the lateral extension of BTC in the multivariate analysis (odds ratio 3.538, 95%; confidence interval 1.151–10.875, *p* = 0.027).

### 3.4. Adverse Events

Adverse events following 24 procedures of POCS-FMB occurred in four patients (16.7%), with three patients (12.5%) developing acute pancreatitis, and one patient (4.2%) developing an infection (acute cholangitis). Severe acute pancreatitis, hemorrhages, perforations, and procedure-related mortalities were not observed, and all cases with adverse events were resolved with conservative treatment (Table 5).

## 4. Discussion

Previous studies reported that BTC has an untoward feature, superficial longitudinal intraductal spread, which is present in 14.6% of patients with cholangiocarcinoma [9,11]. The presence of lateral extension is related to positive resection margins, local recurrences, and poor prognosis after surgical resection. To avoid those unfortunate outcomes of surgery, it is important to identify the accurate disease extent of preoperative BTC. One of the methods to identify the accurate disease extent of BTC is imaging findings, including CT, MRI, ERCP, transpapillary intraductal ultrasonography (IDUS), and POCS. CT and MRI were useful for assessment of the intraductal spread of BTC; the diagnostic accuracies were reported to be 77–92% and 71–80%, respectively [12]. The utility of transpapillary IDUS, one of the ERCP techniques, for the assessment of lateral extension of cholangiocarcinoma was also reported, and the diagnostic accuracy of that varied from 80% to 84% [20]. However, the diagnostic performances of those methods, especially the specificities, were insufficient because it is difficult to distinguish BTC from inflammatory changes by those imagings [13,21]. Moreover, after endoscopic biliary stenting, it is hard to differentiate the hypertrophy of the biliary tract due to BTC from inflammatory changes [22]. In the future, imaging modalities with high specificity may exist. Indeed, the evaluation of surficial spread of gastrointestinal carcinoma was generally performed by gastrointestinal endoscopy with an image-enhanced technique, such as magnifying narrow-band imaging (NBI), and chromoendoscopy [23,24]. Therefore, the visual assessment of POCS might play more important role for the evaluation of the lateral extent of BTC. However, image-enhanced endoscopy except NBI is not currently available, so now another method, such as forceps mapping biopsy, should be needed.

The previous study reported the utility of mapping biopsy techniques in fluoroscopy to evaluate the lateral extension of BTC [14]. Although the specificity was high (100%), the sensitivity was not enough (77.8%). Moreover, it was uncertain whether the biopsy site of that technique, under fluoroscopy without the direct vision of the biliary system, was true or not. Recently, POCS has become a common technique for the diagnosis of indeterminate biliary disease. POCS do not only allows optical direct viewing of the biliary system but also targeted forceps biopsies under the direct vision of the biliary system, with the fluoroscopy as needed. Although the utility of POCS-FMB in the preoperative evaluation of the distal and perihilar margins of BTC was reported, the sensitivity of that was necessarily insufficient [17,19,25]. One reason for this may be that a small amount of specimens was obtained by POCS-FMB. [26]. Some (11.9–52.7%) of the specimens obtained by POCS-FMB were insufficient for histopathological evaluation [25,27]. In our study, the adequate tissue acquisition rate of POCS-FMB was also insufficient (78.1%). This is the most essential reason for decreasing the diagnostic accuracy for the preoperative assessment for the lateral extension of BTC. Indeed, if only the adequate specimens were evaluated in the study, the sensitivity, specificity, and accuracy were 53.8% (7/13), 90.2% (46/51), and 82.8% (53/64), respectively. Both larger capacity forceps and POCS with a larger diameter working channel where the forceps can be inserted into might improve the diagnostic performance of POCS-FMB in the near future [28]. Multiple biopsies might be also helpful to improve the diagnostic accuracy of POCS-FMB.

Although the study has several limitations, such as retrospective analysis of the Japanese population with a small participant sample in a single center, it is the first report that revealed the diagnostic performance of POCS-FMB for the evaluation of lateral extension of BTC by the biopsy sites. Moreover, the factor affecting the accuracy of POCS-FMB is evaluated with consideration of the biopsy site. Understanding the negative factors affecting the diagnostic performance of POCS-FMB may lead to the improvement of diagnostic accuracy. In multivariate logistic regression analyses, we found that the biopsy from the bifurcation of the biliary tract was a positive affecting factor for the diagnostic accuracy of POCS-FMB. Compared to POCS-FMB from the bifurcation of biliary tracts, biopsies from the lumen of biliary tracts, such as the distal bile duct, the perihilar bile duct, and the left and right hepatic ducts, were technically difficult; forceps contact might detach the biliary epithelium from biliary tract before the biopsy is undergone. Therefore, the specimen with pathologically assessable biliary epithelium may be lost. Furthermore, POCS-FMB from the lumen of biliary tracts was slippery, and could not be performed from the desired site; therefore, contamination of specimens from other sites might occur. In clinical practice, careful and accurate procedure of POCS-FMB is required to obtain the specimens from the desired lumen of biliary tracts. A more flexible cholangioscope with forceps that prevents displacement from the targeted site might help to improve the diagnostic accuracy of POCS-FMB for the lumen of biliary tracts.

The study has some limitations. First, this was a retrospective analysis of an Asian population with a small number of cases limited to the surgical population in a single institute. The non-surgical population in patients who received POCS-FMB was also excluded. As a result, the study included a widely varied patient population from a single institute, and the total number of patients analyzed was relatively small. This fact might lead to few data about patients with poor performance status. However, POCS-FMB is primarily indicated for patients with good performance status who are candidates for surgery. Moreover, the study included only four patients with perihilar cholangiocarcinoma, whose assessment for lateral extension is generally required for curative resection. Although the study mainly included patients with distal cholangiocarcinoma, the frequency of their lateral extension is not ignored. Therefore, it is also essential to evaluate the diagnostic accuracy of POCS-FMB for distal cholangiocarcinoma. Second, the specimens obtained from biopsy sites that were finally diagnosed through clinical follow-up data were also included in the study. As a result, it was uncertain whether the biopsy sites with negative pathological findings were truly benign or not. However, to evaluate the biopsy site with negative pathological findings, clinical follow-up data were required since evaluation of the site outside the resected specimen was necessary. Otherwise, our study might be limited to patients who underwent curative surgical resection for BTC, and this was a critical selection bias. Third, in patients who received adjuvant therapy or radiation therapy, it was uncertain whether the final diagnosis for the lateral extension of BTC was correct or not. Moreover, other unknown candidate factors affecting the accuracy of POCS-FMB may exist, and we might not evaluate them. Needless to say, a prospective long-term study including a larger number of patients in multiple centers is required to investigate the diagnostic utility of POCS-FMB more.

## 5. Conclusions

The diagnostic accuracy of POCS-FMB for lateral extension of BTC was only 63.1% in our retrospective study. The most essential reason for that was the inadequate tissue acquisition rate of POCS-FMB (78.1%). POCS-FMB from the bifurcation of biliary tracts was a significant factor affecting high diagnostic accuracy.

## Figures and Tables

**Table 1:** Baseline characteristics of study participants.

	Patients with BTC (*n* = 23)
Age, years	72 (56–84)
Sex, male/female	16/7
Location of BTC (*n* = 24)	
Distal	16
Perihilar	4
Cystic duct	3
Gall bladder	1
Length of stricture, mm (*n* = 24)	16.8 (4.8–45.6)
Acute cholangitis (presence/absence) (*n* = 24)	22/2
Total bilirubin, mg/dL	1.6 (0.5–14.8)
Tumor marker	
CEA, ng/mL	2.6 (0.8–8.3)
CA19-9, U/mL	35.2 (6.4–9052.0)
Macroscopic type (*n* = 24)	
Papillary type	5
Nodular type	13
Flat type	6
Histological differentiation (*n* = 24)	
Well differentiated tubular adenocarcinoma	12
Moderately differentiated tubular adenocarcinoma	7
Poorly differentiated adenocarcinoma	3
Papillary adenocarcinoma	2
TNM classification/Stage (*n* = 24)	
Distal	
T category 1/2/3/4	2/8/6/0
N category 0/1	4/12
M category 0/1	16/0
Stage IA/IB/IIA/IIB/III/IV	2/4/6/4/0/0
Perihilar	
T category 1/2/3/4	0/4/0/0
N category 0/1	1/3
M category 0/1	4/0
Stage I/II/IIIA/IIIB/IVA/IVB	0/3/0/1/0/0
Gall bladder/Cystic duct	
T category 1/2/3/4	0/2/2/0
N category 0/1	4/0
M category 0/1	4/0
Stage I/II/IIIA/IIIB/IVA/IVB	0/2/2/0/0/0

Values are presented as number or median (range). Abbreviations: BTC, biliary tract cancer; CEA, carcinoembryonic antigen; CA19-9, carbohydrate antigen 19-9.

**Table 2:** Procedure of peroral cholangioscopy-guided forceps mapping biopsy.

	POCS (*n* = 24)
Procedure time, minutes	85.0 (57–124)
EST, previous/with/without	7/14/3
Number of biopsy, times	2 (1–4)

Values are presented as number or median (range). Abbreviations: POCS, peroral cholangioscopy; EST, endoscopic sphincterotomy.

**Table 3:** Diagnostic performance of peroral cholangioscopy-guided forceps mapping biopsy.

Biopsy Site	Sensitivity, %	Specificity, %	Accuracy, %
Total	53.8 (7/13)	63.9 (46/71)	63.1 (53/84)
Distal bile duct	50.0 (1/2)	40.0 (2/5)	42.9 (3/7)
Junction of the cystic duct	50.0 (2/4)	100 (4/4)	75.0 (6/8)
Perihilar bile duct	0 (0/1)	0 (0/2)	0 (0/3)
Confluence of the hepatic duct	66.7 (2/3)	70.6 (12/17)	70.0 (14/20)
Right hepatic duct	0 (0/1)	50.0 (1/2)	33.3 (1/3)
Confluence of the anterior and posterior segmental ducts	100 (1/1)	58.3 (7/12)	61.5 (8/13)
Confluence of B5 and B8 segmental ducts	100 (1/1)	50.0 (2/4)	60.0 (3/5)
Confluence of B6 and B7 segmental ducts	-	100 (2/2)	100 (2/2)
Left hepatic duct	-	60.0 (3/5)	60.0 (3/5)
B4 confluence	-	50.0 (4/8)	50.0 (4/8)
Confluence of B2 and B3 segmental ducts	-	90.0 (9/10)	90.0 (9/10)

**Table 4:** Factors affecting the accuracy of peroral cholangioscopy-guided forceps mapping biopsy to identify the lateral extension of biliary tract cancer.

**Univariate Analyses**			
**Subgroup**	**Odds Ratio**	**95% CI**	***p* Value**
Age, <75 years or ≥75 years	0.554	0.226–1.357	0.196
Sex, male or female	1.744	0.700–4.344	0.232
Location of biliary tract cancer, distal or not distal	2.411	0.904–6.434	0.079
Length of stricture of biliary tract, <15mm or ≥15mm	0.909	0.369–2.237	0.835
Macroscopic type, flat type or not-flat type	0.669	0.232–1.927	0.669
histological differentiation (tubular adenocarcinoma or not)	0.734	0.229–2.353	0.603
T category, T<3 or ≥3	0.726	0.286–1.844	0.500
Cholangitis, presence or absence	0.972	0.216–4.380	0.971
T-Bil, <1.5 mg/dl or ≥1.5 mg/dl	0.584	0.239–1.429	0.239
CEA, < 5.0 ng/mL or ≥5.0 ng/mL	1.496	0.519–4.311	0.456
CA19-9, U/mL <35 U/mL or ≥35 U/mL	0.809	0.331–1.978	0.642
Procedure time, <75 min or ≥75 min	1.003	0.348–2.895	0.995
EST or non-EST	4.000	0.923–17.340	0.064
Number of biopsy, <2 or ≥2	0.841	0.300–2.357	0.743
Previous biliary stenting, presence or absence	0.682	0.280–1.660	0.399
Biopsy site			
Intrahepatic bile duct or extrahepatic bile duct	1.525	0.619–3.755	0.359
Bifurcation of biliary tract or not	3.614	1.223–10.678	0.020
Right side or left side	0.681	0.201–2.307	0.537
**Multivariate Analyses**			
**Subgroup**	**Odds Ratio**	**95% CI**	***p* Value**
Location of biliary tract cancer, distal or not distal	1.974	0.695–5.604	0.201
EST or non-EST	3.924	0.849–18.130	0.080
Biopsy site, bifurcation or not	3.538	1.151–10.875	0.027

Abbreviations: CI, confidence interval; T-Bil, total bilirubin; CEA, carcinoembryonic antigen; CA19-9, carbohydrate antigen; EST, endoscopic sphincterotomy. *p* Value: Logistic regression model.

**Table 5:** The adverse event of peroral cholangioscopy-guided forceps mapping biopsy.

Adverse Event	POCS-Guided Forceps Mapping Biopsy (*n* = 24)
Pancreatitis	12.5% (3/24)
Bleeding	0
Infection	4.2% (1/24)
Perforation	0
Cardiac	0
Pulmonary	0
Medication reaction	0
Other	0
Overall	16.7% (4/24)

Abbreviations: POCS, peroral cholangioscopy.

## Data Availability

Data sharing is not applicable to this article. The data are not publicly available due to restrictions of privacy and ethics.
